# The Toxin Diversity, Cytotoxicity, and Enzymatic Activity of Cape Cobra (*Naja nivea*) Venom

**DOI:** 10.3390/toxins16100438

**Published:** 2024-10-11

**Authors:** Tim Lüddecke, Ignazio Avella, Maik Damm, Lennart Schulte, Johanna Eichberg, Kornelia Hardes, Susanne Schiffmann, Marina Henke, Thomas Timm, Günter Lochnit, Andreas Vilcinskas

**Affiliations:** 1 Animal Venomics Lab, Fraunhofer Institute for Molecular Biology and Applied Ecology IME, Ohlebergsweg 12, 35392 Giessen, Germany; ignazio.avella@ime.fraunhofer.de (I.A.); maik.damm@outlook.de (M.D.); lennart.schulte@ime.fraunhofer.de (L.S.); 2 Department of Bioresources, Fraunhofer Institute for Molecular Biology and Applied Ecology IME, Ohlebergsweg 12, 35392 Giessen, Germany; johanna.eichberg@ime.fraunhofer.de (J.E.); kornelia.hardes@ime.fraunhofer.de (K.H.); andreas.vilcinskas@ime.fraunhofer.de (A.V.); 3 LOEWE Centre for Translational Biodiversity Genomics (LOEWE-TBG), Senckenberganlage 25, 60325 Frankfurt am Main, Germany; susanne.schiffmann@itmp.fraunhofer.de (S.S.); marina.henke@itmp.fraunhofer.de (M.H.); 4 Institute for Insect Biotechnology, Justus Liebig University Giessen, Heinrich-Buff-Ring 26–32, 35392 Giessen, Germany; 5 BMBF Junior Research Group in Infection Research “ASCRIBE”, Ohlebergsweg 12, 35392 Giessen, Germany; 6 Fraunhofer Institute for Translational Medicine and Pharmacology ITMP, 60596 Frankfurt am Main, Germany; 7 Institute for Biochemistry, Justus Liebig University Giessen, Friedrichstrasse 24, 35392 Giessen, Germany; thomas.timm@biochemie.med.uni-giessen.de (T.T.); guenter.lochnit@biochemie.med.uni-giessen.de (G.L.)

**Keywords:** snakebite, Elapidae, bioassays, proteomics, venomics, 3FTx, Africa, *Uraeus*

## Abstract

“True” cobras (genus *Naja*) are among the venomous snakes most frequently involved in snakebite accidents in Africa and Asia. The Cape cobra (*Naja nivea*) is one of the African cobras of highest medical importance, but much remains to be learned about its venom. Here, we used a shotgun proteomics approach to better understand the qualitative composition of *N. nivea* venom and tested its cytotoxicity and protease activity as well as its effect on intracellular Ca^2+^ release and NO synthesis. We identified 156 venom components representing 17 protein families, with the dominant ones being three-finger toxins, mostly of the short-chain type. Two-thirds of the three-finger toxin entries identified were assigned as cytotoxins, while the remainder were categorized as neurotoxins, including short-chain, long-chain, and ancestral three-finger toxins. We also identified snake venom metalloproteinases and members of CRISP, l-amino acid oxidase, and other families. Protease activity and its effect on intracellular Ca^2+^ release and NO synthesis were low. Phospholipase A_2_ activity was surprisingly high, despite this toxin family being marginally recovered in the analyzed venom. Cytotoxicity was relevant only at higher venom concentrations, with macrophage and neuroblastoma cell lines showing the lowest viability. These results are in line with the predominantly neurotoxic envenomation symptoms caused by Cape cobra bites. The present overview of the qualitatively complex and functionally intriguing venom of *N. nivea* may provide insights into the pathobiochemistry of this species’ venom.

## 1. Introduction

Snakebites are a frequent cause of death and morbidity in developing countries especially in Africa and Asia, and the socioeconomic consequences can be devastating when young working men (the main breadwinners for most families) are envenomated [1,2,3,4]. The high incidence and significant impact of snakebites in many of the economically weakest societies in the world have promoted interest in the composition of snake venoms [3] in order to facilitate the development of more effective therapeutics and treatment protocols [5,6,7,8]. This area of research has recently been accelerated by the emergence of disruptive technologies from the biotechnological sector [9,10,11,12]. It is important to understand the molecular profile of snake venoms, including the activity of all their proteins and peptides [13,14]. Proteomics has therefore been used to investigate the venom composition of several medically significant snake species [15,16,17,18,19,20].

**Figure 1:**
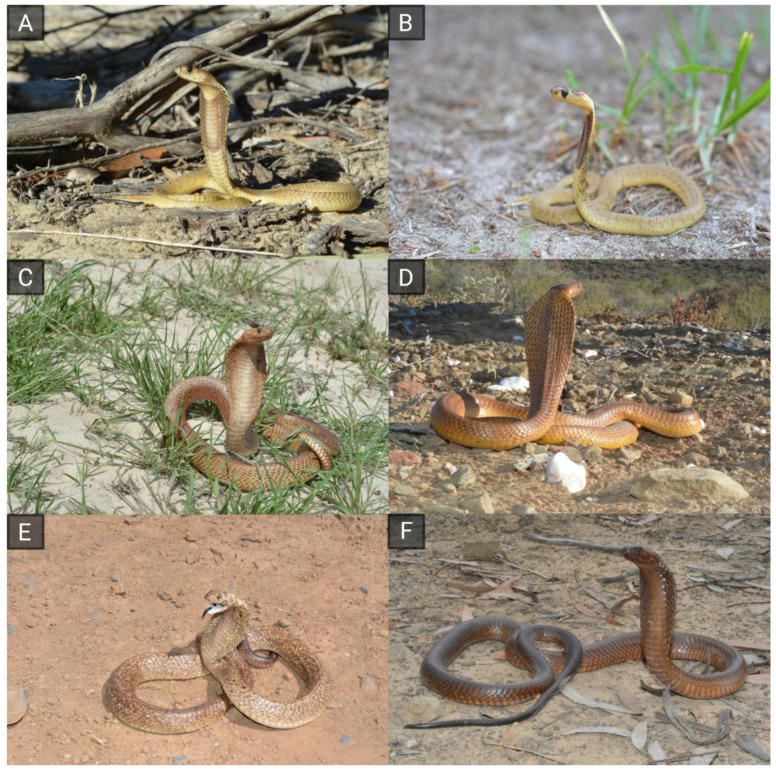
Phenotypic variation and hooding behavior in the Cape cobra (*Naja nivea*). The top row shows juveniles (**A**,**B**); adults are shown in middle and bottom rows (**C**–**F**). Specimens in (**C**,**D**) display the widespread yellowish to brown coloration, (**E**) shows a speckled specimen, and a dark brown variety is shown in (**F**). Images by Andries Cilliers.

Approximately 400 venomous snake species are currently recognized as medically important by the World Health Organization [21]. Among these, “true” cobras of the genus *Naja* deserve special attention. They are arguably among the most famous venomous snakes in the world, and their distinctive hooding behavior has made them widely recognizable to the general public. The genus *Naja* belongs to the family Elapidae and features ~30 species distributed across Africa and Asia [22,23]. Together with the genera *Echis* and *Daboia* (both belonging to the family Viperidae), the genus *Naja* is one of the genera leading causes of snakebites in the Old World [2]. *Naja* venoms are of severe toxicity, and virtually all *Naja* species are able to cause lethal envenomation in humans. The principal lethal components of *Naja* venoms are neurotoxins, but they also contain cytotoxins that cause tissue damage [24]. Considering the high medical relevance of cobra venoms, their effects and properties have been widely investigated by means of different approaches [24,25,26,27,28,29,30,31,32]. Venom research has focused on species responsible for the highest incidence of snakebites in different regions, such as the Indian cobra (*Naja naja*) in Asia [2,26]. While a series of studies investigated the venom of the Cape cobra (*Naja nivea*), this species has overall received less attention than several other true cobras. This representative of the subgenus *Uraeus*, exclusively comprising African non-spitting cobras (e.g., *Naja haje*, *Naja annulifera*), occurs in Namibia, Botswana, South Africa, and Lesotho [15,20,21]. It is one of the smaller African cobras (maximum length 150 cm), although larger specimens have been recorded [33,34,35,36] and often features a distinctive yellowish coloration that makes it very popular in the pet trade [37] (see Figure 1). *N. nivea* bites cause potentially lethal neurotoxic symptoms, particularly areflexic flaccid paralysis requiring prolonged ventilation and antivenom therapy, usually commencing less than 3 h post bite [38]. Considering the mainly neurotoxic symptoms generally arising after *N. nivea* envenoming, the potential of this species’ venom for causing different effects has scarcely been explored. The *N. nivea* venom shows high potency against mammalian targets (e.g., LD_50_ = 0.41–0.75 µg/g; murine) [17,39]. 

The Cape cobra played an important role in the establishment of the first functional snake venom gland organoids, and several individual toxins have already been isolated from the venom of this species [40,41,42,43,44]. However, to date, only a few studies have investigated the composition of Cape cobra venom using proteomics methods [25,27,45,46]. The composition and biological activities of snake venom may vary at intraspecific levels depending on factors like sex of the analyzed individuals, locality of origin, and seasonality, so it is important to analyze different specimens to gain a full understanding of the dynamic and variable venom components [47,48,49]. In the present study, we thus provide a qualitative profile of *N. nivea* venom from South Africa using a shotgun proteomics approach and investigate its bioactivity profile. Our results provide novel data to further support global efforts toward the development of more effective therapeutic tools to treat envenomation caused by Cape cobras.

## 2. Results

### 2.1. Venom Profiling by 1D-SDS-PAGE

**Figure 2:**
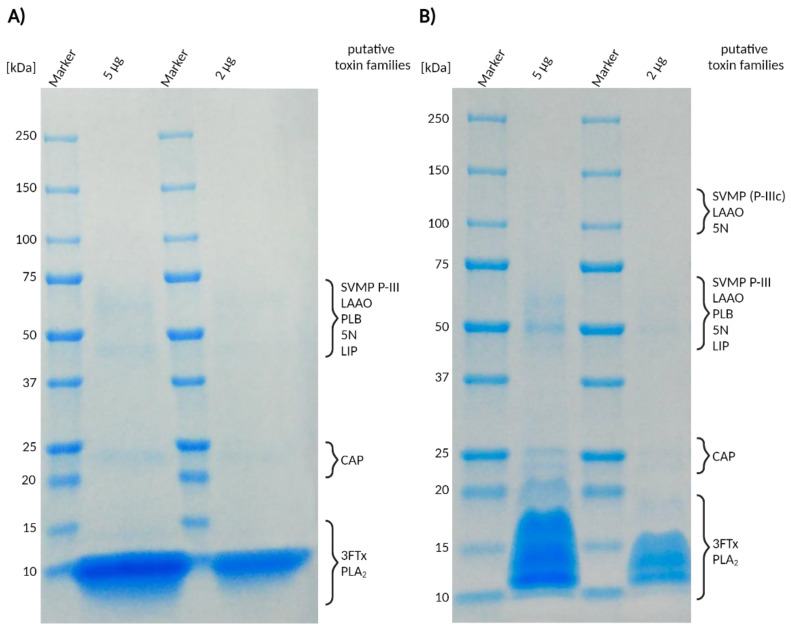
One-dimensional SDS-PAGE of *Naja nivea* venom under (**A**) reduced and (**B**) non-reduced conditions. Shown are stained protein bands of 5 µg and 2 µg venom. Putative toxin classes matching the molecular weight of the retrieved bands are assigned based on their known sizes. Abbreviations: svMP P-III = snake venom metalloproteinases of class P-III; LAAO = l-amino acid oxidase; PLA_2_ = phospholipase A_2_; PLB = phospholipase B; CE = carboxylesterase; 5N = 5′-nucleotidase; CRISP = cysteine-rich secretory proteins; 3FTx = three-finger toxins.

SDS-PAGE analysis was run under reducing and non-reducing conditions (see Figure 2). Under both conditions, the gels were characterized by the absence of intense bands at high molecular ranges. In contrast, the overwhelming majority of identified bands were present at lower molecular ranges (<25 kDa). The analysis under both SDS-PAGE conditions revealed similar banding patterns. Overall, the SDS-PAGE profiles suggest that the venom of N. nivea is strongly dominated by small venom proteins (<15 kDa). Bands at apparent masses for important snake venom toxin families known to occur in the genus *Naja* imply that a range of those are present, albeit in lower amounts. This includes snake venom metalloproteinases (svMPs), l-amino acid oxidase (LAAO), and cysteine-rich secretory proteins (CRISPs). However, in contrast to those, intense bands are retrieved under both reducing and non-reducing conditions (10 kDa reduced and 10–20 kDa in non-reduced), matching sizes for three-finger toxins (3FTxs) and phospholipase A_2_ (PLA_2_). These are often found in venoms of the Elapidae family, including members of the genus *Naja* [24,40,41,50,51,52]. The disappearance of the 10–20 kDa band (non-reduced) and formation of a 10 kDa band (reduced) suggest the dismantling of potential covalent oligomers, as they are known for only a few snake 3FTxs [53,54]. Since SDS-PAGE profiling provides only limited information on protein sizes within the sample, and any family assignments made are purely putative, additional mass spectrometric evidence is necessary to fully elucidate the venom profile of the analyzed venom.

### 2.2. Shotgun Venom Proteomics

To support the protein assignments made via 1D-SDS-PAGE, we proceeded to perform a mass spectrometric validation of our findings. Using a shotgun proteomics strategy, we detected 168 proteins representing 23 distinct families (Table S1) across our entire sample. After removal of potential non-venom components from the analysis, our final dataset contained 156 venom components that could be assigned to 17 protein families. Most of the protein families were represented by more than one protein, and all had previously been found in snake venoms.

**Figure 3:**
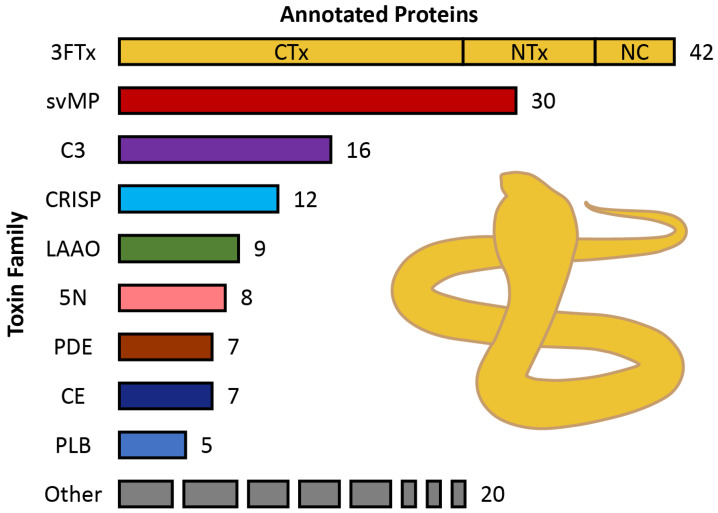
Bar chart depicting toxin diversity in *Naja nivea* venom. The numbers refer to the variety of venom proteins detected within each protein family, thus providing a snapshot of the toxin diversity within *N. nivea* venom. The most diverse component family was three-finger toxins (3FTxs), with cytotoxins (CTx, 26 entries), neurotoxins (NTx, 10 entries) including 1 muscarinic toxin, and non-conventional neurotoxins (NC, 6 entries). The second most diverse family was snake venom metalloproteinases (svMP), followed by venom complement C3 proteins, cysteine-rich secretory proteins (CRISP), l-amino acid oxidases (LAAO), 5′ nucleotidases (5N), carboxylesterases (CE), phosphodiesterases, and phospholipase B proteins (PLB). The less diverse families (<5 components) are grouped as “Other”.

The qualitative composition of *N. nivea* venom is summarized in Figure 3. The most diverse family in the *N. nivea* venom proteome was the three-finger toxin (3FTx) family, with 42 members spanning three major subgroups (Table 1). Specifically, we identified six non-conventional (NC) neurotoxic 3FTxs. Most of these displayed similarities with other *Naja* NC 3FTxs, including *N. nivea* (P25680), *N. haje* (P01401), *N. melanoleuca* (P01400), and *N. naja* (P29181), but also with Bucandin (P81782), a presynaptic neurotoxin from the Malayan krait *Bungarus candidus*. We identified six short-chain and three long-chain neurotoxic 3FTxs, with the short neurotoxin 2 (P01423) and long neurotoxin 1 (P01390), both from *Naja nivea*, showing the highest MASCOT score for those subgroups, respectively. While most 3FTxs displayed similarity to known *Naja* toxins, one of the proteins was similar to a muscarinic m-1 toxin (P60234) from the Eastern green mamba *Dendroaspis angusticeps*. 

However, the most diverse subgroup was the cytotoxic 3FTxs (CTxs), which are also referred to as cardiotoxic 3FTxs with 26 members being homologs to toxins of spitting and non-spitting cobras, as *N. nivea* [55]. The six CTxs with the highest MASCOT score were CTxs 1, 2, and 3 (*N. nivea*; P01456, P01463, and P01458) and CTxs 2, 4, and 10 (*N. annulifera*; P01461, P01462, and P01453). In total, one third of the 3FTxs in *N. nivea* venom were therefore assigned as neurotoxins (16 members) and two thirds as cytotoxins (26 members), together accounting for 26.9% of all Cape cobra venom components (Table 1).

The second most diverse components were svMPs, which belong to the M12B family of metalloproteinases. We identified 30 svMPs, corresponding to 19.2% of the venom components. We also identified 16 venom complement C3 proteins (10.3%) and 12 CRISP members of the cysteine-rich secretory protein/antigen 5/pathogenesis-related protein (CAP) superfamily, representing 7.7% of the venom components. We detected nine LAAOs (5.8%), eight 5′ nucleotidases (5Ns, 5.1%), seven carboxylesterases (CEs, 4.5%), and seven phosphodiesterases (PDEs, 4.5%), as well as five phospholipase B proteins (PLBs, 3.2%).

The *N. nivea* venom sample analyzed also contained various less diverse toxin families with only 1–4 components, which are shown as “Other” in Figure 3. The most diverse of these were the aminopeptidases of the M1 family and endonuclease families, each represented by four members (2.6% of the protein diversity). The nerve growth factor β, snake venom serine protease (S1 family), and PLA_2_ were each represented by three members (1.9%). Two of the PLA_2_ proteins were basic and one acidic. One member was detected for the Kunitz, Ohanin/Vespryn, and vascular endothelial growth factor families, together accounting for 0.6% of the N. nivea venom analyzed.

### 2.3. Bioactivity Profiling

**Figure 4:**
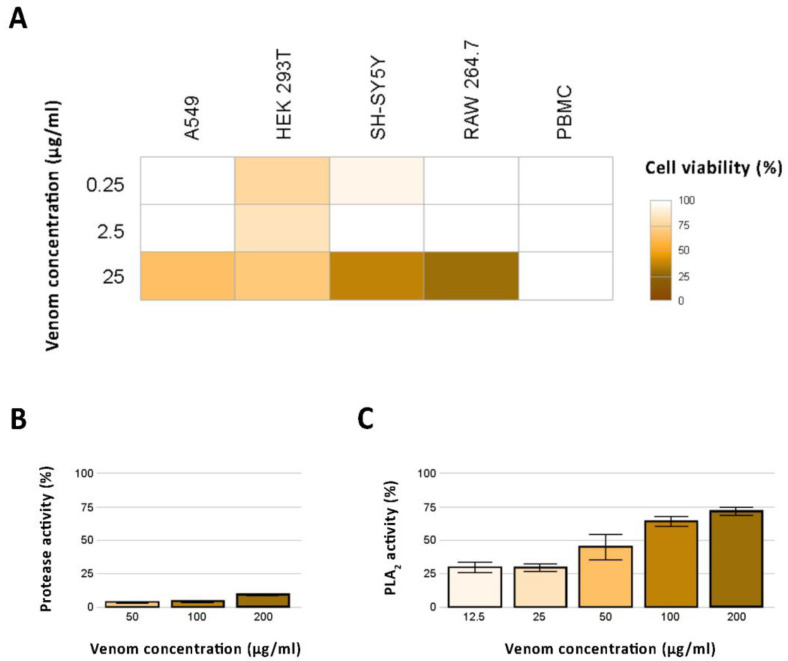
In vitro effects of *N. nivea* venom. (**A**) The heatmap shows the cytotoxic effect of the analyzed *N. nivea* venom on the viability of the five tested cell lines (A549, HEK 293T, SH-SY5Y, RAW 264.7, PBMC). (**B**) Protease and (**C**) phospholipase A_2_ activity. The bar charts illustrate the photometrically determined protease and PLA_2_ activity of *N. nivea* venom at three concentrations relative to the trypsin control, and at five concentrations relative to the PLA_2_ stock solution provided with the assay kit, respectively.

Considering the diversity of cytotoxins found in the produced *N. nivea* venom proteome, we first explored the cytotoxic effects of this species’ venom across four different mammalian cell lines (murine, RAW 264.7; human, A549, HEK 293T, and SH-SY5Y) and primary human peripheral blood mononuclear cells (PBMCs). Evident cytotoxicity was detectable almost exclusively at the highest venom concentration, with cell viability ranging from 29% (RAW 264.7) to 70% (HEK 293T) at 25 µg/mL. Virtually no cytotoxicity was detected at concentrations of 2.5 and 0.25 µg/mL across all tested cell lines but HEK 293T, for which viability ranged from 78% (0.25 µg/mL) to 70% (25 µg/mL). No cytotoxic effect was detected on PBMCs. Results of the cytotoxicity assays are presented in Figure 4A.

Furthermore, we assessed the protease and PLA_2_ activity of the analyzed *N. nivea* venom. The venom showed low levels of protease activity across all tested concentrations, ranging from 5% to 10% in a concentration-dependent manner (Figure 4B). Also, the PLA_2_ activity varied in a positive concentration-dependent manner but was considerably more remarkable, ranging from 31% to 73% (Figure 4C). Finally, aiming to test whether *N. nivea* venom impacts cell–cell communication and inflammation, we investigated its effect on intracellular Ca^2+^ release in HEK 293T and SH-SY5Y cells, as well as on NO levels in RAW 264.7 cells. Our analysis revealed no apparent effect of *N. nivea* venom on either of the tested targets. Additional information on the performed bioassays is provided in Tables S2–S7.

## 3. Discussion

Through the application of a shotgun proteomics approach paired with one-dimensional SDS-PAGE profiling, we determined the qualitative profile of *N. nivea* venom components (Figure 2 and Figure 3). This provided deeper insights into the putative biological diversity of venom proteins and the medical consequences of envenomation by Cape cobras, which is characterized by the rapid onset of neurotoxic effects. In some verified bites, life-threatening flaccid paralysis developed within 3 h, requiring a combination of prolonged ventilation and antivenom therapy [38]. Untreated bites are often fatal, but even after prompt treatment, it may still be necessary for patients to remain in-hospital for several days [38]. We have identified several components that may be responsible for the extreme and prolonged neurotoxicity of *N. nivea* venom.

Fatalities caused by elapid snakebites often reflect the activity of 3FTxs and PLA_2_ [56], two of the largest protein families found in elapid snake venoms [57]. The 3FTxs contain the characteristic, three-finger fold motif, comprising three loops that protrude from the protein core to form a finger-like structure [56]. Plesiotypic 3FTxs bind to neuromuscular α1 nicotinic acetylcholine receptors. Such ancestral toxins are often described as “weak neurotoxins” or non-conventional due to their low affinity for human receptors and the consequently mild neurotoxic symptoms they typically elicit in humans [55,56]. However, their toxicity in reptiles and birds is significantly higher, and the term “weak neurotoxins” is therefore likely inadequate [58]. Elapids have also evolved a large repertoire of apotypic 3FTxs through the loss of functionally constraining cysteine residues [56,59]. Our exploratory examination of the *N. nivea* venom profile by means of SDS-PAGE under reducing- and non-reducing conditions showed that Cape cobra venom is predominantly composed of smaller-sized proteins below 15 kDa. This banding pattern is to be expected by venoms predominantly composed of 3FTxs such as those of the genus *Naja*.

Furthermore, we conducted a shotgun proteomics investigation of the *N. nivea* venom (Table 1, Figure 3, and Supplementary Table S1). This analysis supported our initial observations and revealed that 3FTxs are indeed the principal components of *N. nivea* venom. Of these, two thirds were identified as cytotoxic 3FTxs, with the remainder classified instead as putative neurotoxins. Seven of the seemingly neurotoxic 3FTxs are α-neurotoxins that potently interfere with human α1 nicotinic acetylcholine receptors and contribute to the lethality as in other cobra venoms [60]. We also found six NC 3FTxs, which can cause mild neurotoxic effects in humans [61]. While the overwhelming majority of identified 3FTxs with putative neurotoxic effects displayed similarities with known toxins previously described from *Naja* venoms (e.g., P25680, P01390, P01423), some of these resembled non-*Naja* elapid toxins. These include one toxin similar to bucandin from the Malayan krait *B. candidus* and one similar to an uncharacterized toxin from the death adder *Acanthophis wellsi* (R4G2D8). Remarkably, one of the short-chain 3FTxs was found to be similar to a muscarinic m-1 toxin (P60234) from the Eastern green mamba *D. angusticeps*, which binds irreversibly and with high specificity to M1 (CHRM1) muscarinic acetylcholine receptors [62]. This prevents the binding of receptor antagonists and downstream signaling for extended time periods, potentially explaining the long-lasting neurotoxic effects of *N. nivea* envenomation.

Despite the medical importance of the Cape cobra, the composition of its venom has only recently been assessed using proteomics [25,27,45,46]. The available proteomics studies on *N. nivea* venom have been analyzed based on different workflows and (semi)-quantification (see Table 2). Some differences in the results are therefore anticipated, due to discrepancies in the experimental designs applied, such as databases used for annotation and technological strengths and weaknesses of the different mass spectrometry protocols [63]. Nonetheless, consistent with previous works, we identified 3FTxs as the most abundant and diverse components of *N. nivea* venom. Cytotoxins were generally more diverse than neurotoxins [25,27,45,46], which were identified as a lower abundant fraction of the 3FTxs, consistent with the venom compositions of some other *Naja* species [25,27,45]. The lower diversity of neurotoxic compared to cytotoxic 3FTxs may reflect the potency and target redundancy of the neurotoxins. A handful of highly potent neurotoxic components are sufficient to ensure the rapid onset of paralysis, and an extension of this chemical arsenal would therefore likely be unnecessary in a scenario where venom is primarily used for prey subjugation.

The appearance of a band around 10 kDa that is smaller under reducing conditions and the disappearance of a band >15 kDa that is larger (non-reduced) indicate that structuring disulfide bonds might have been cleaved and monomers were formed. Even if 3FTxs are mostly known as monomeric venom compounds, a few dimeric 3FTxs were discovered in different snake venoms recently. While Haditoxin (*Ophiophagus hannah*) and fulditoxin (*Micrurus fulvis*) are non-covalent homodimeric 3FTxs, covalent 3FTx complexes have been identified in the venom of the closely related *Naja kauthia* (α-cobratoxin homodimer and heterodimers), as well as two rear-fanged snakes *Bioga irregularis* (irditoxin) and *Spilotes sulphureus* (sulditoxin) [53,54,64,65]. McFarlane et al. (2024) hypothesized about the existence of covalent 3FTxs in the venom of *N. nivea*. By using a different venom pool, they showed a comparable banding pattern in reduced and non-reduced venom SDS PAGEs [46]. By LC-MS intact mass profiling, the authors listed over 20 masses in the range of potential NTxs and CTxs, which could mathematically form dimers of 12 to 16 kDa, corresponding to the reported bands. The 3FTxs with the highest MASCOT score we identified in our *N. nivea* venom sample were identical to the study of McFarlane et al., where they also formed the highest abundancies by NSAF quantification. This includes the cytotoxins 1, 2, and 3 (P01456, P01463, P01458); the long neurotoxin 1 (P01390); and cytotoxins 2 and 10 (P01462, P01453) of *Naja annulifera*, which belongs to the same subgenus *Uraeus,* leading to assumptions that the covalent 3FTxs of *N. nivea* can be cytotoxic, neurotoxic, or even a combination of cyto- and neurotoxic 3FTxs.

The work of Nguyen et al. (2022) [22] presents a higher protein family diversity, potentially due to their use of an in-house venom gland transcriptome as a database for protein identification. Additional retrieved venom components included hyaluronidases (HYALs), cystatin (CYS), C-type lectins and lectin-related proteins (CTL), and disintegrins (DIs), each accounting for less than 0.01% of the produced *N. nivea* venom proteome. Regarding the other toxin families, exclusively the study of Tan et al. (2022) [45] reported the absence of PLA_2_ in Cape cobra venom. This discrepancy underpins the importance of investigating different samples of a species using different methods, in order to avoid generalized assumptions stemming from limited sample sizes and analytical platforms. The *N. nivea* venom investigated by Kazandjian et al. (2021) showed only five toxin families in total, most likely as a consequence of their purely top-down-based workflow, known for its limitations when it comes to the analysis of low-abundance analytes with high molecular mass (e.g., svMP, LAAO, PDE, 5N) [27,66,67]. That said, it cannot be excluded that venom variation is at play in Cape cobras. This phenomenon has been reported widely across the snake kingdom and it is well known that venom profiles can differ tremendously between individuals, based on factors such as locality, sex, and life history stages [68]. That said, this subject has not been studied in Cape cobras sufficiently and we recommend that future studies investigate it. Interestingly, the analysis of coloration phenotypes in the context of venom variation has recently started to gain traction, but the results are inconclusive [69,70,71]. Facing the large phenotypic variability of Cape cobras (see Figure 1), it would be interesting to also investigate this particularity in the future.

Interestingly, all studies aiming to characterize *N. nivea* venom (including the present one) detected the lowest amounts of PLA_2_, in contrast to the venoms of other African and Asian members of the genus *Naja* [25,27,45]. Although proteomic studies suggest that PLA_2_ proteins are underrepresented in non-spitting cobras of the subgenus *Uraeus*, insights from bioactivity-guided experiments partially contradict these findings. For example, our PLA2 activity assay detected considerable concentration-dependent effects, between 30.95% (lowest concentration) and 72.56% (highest concentration) in comparison to crude bee venom used as a positive control. Likewise, PLA_2_ activities of 1–30 U/mg were reported in 11 individual specimens of *N. nivea*, as well as 65 U/mg in *N. haje*, 62 U/mg in *N. melanoleuca* (African non-spitting cobras), 125 U/mg in *N. nigricollis* (African spitting cobra), and 70 U/mg in *Hemachatus haemachatus* (African spitting elapid, but not a “true” cobra) [72]. Although these data agree with the proteomics-based theory of the higher relevance of PLA_2_ in spitting cobra venoms, they also show that the extreme variability recently reported in several animal venoms may play an important role, and that several non-spitting cobra venoms may feature ~25% (*N. nivea*) or even ~50% (e.g., *N. haje* and *N. melanoleuca*) of the PLA_2_ activity present in spitting cobras (*N. nigricollis*) [72]. This highlights the need to investigate different populations from each venomous species and generate multiple datasets to account for the phenomenon of intraspecific venom variability in snakes and other venomous taxa. 

In light of the detected diversity of components in the analyzed *N. nivea* venom, we investigated its function by testing its protease activity and role in intracellular Ca^2+^ release and NO synthesis. Furthermore, considering the high diversity of cytotoxic 3FTxs observed, we assessed the venom’s cytotoxic activity on a diverse array of mammalian cells. Despite finding minuscule effects for the protease activity and Ca^2+^ and NO assays (Figure 4B and Tables S4–S6), we observed the presence of cytotoxic activity. Indeed, at the highest concentration (i.e., 25 µg/mL), *N. nivea* decreased the viability of most tested cells (Figure 4A), whereas lower concentrations had virtually no cytotoxic effect, and the PBMCs were completely unaffected. The highest venom concentration caused a decrease in cell viability of approximately 30% in the epithelial-like kidney cells (HEK 293T) and epithelial lung cells (A549). These findings appear concordant with a previous cell viability assay performed by Nguyen and colleagues on human N/TERT keratinocytes, and suggest that *N. nivea* venom generally exerts a weak cytotoxic effect on epithelial cells [25]. Nonetheless, our bioassays recovered a considerable cytotoxicity of *N. nivea* venom at a concentration of 25 µg/mL on SH-SY5Y neuroblastoma cells, reducing their viability to approximately 37%. An even more marked decrease in viability was detected in the macrophage cell line RAW 264.7 (29% viability at venom concentration of 25 µg/mL). As a decrease in macrophage number may play a role in impairing the inflammatory response and delaying wound healing [73,74], it may increase the risk of secondary infection, and should thus be considered when treating *N. nivea* snakebites. Although infection following bites of most snakes is not particularly common, snake venoms have recently been shown to contain complex microbiomes, and thus the aspect of microbial infection post envenoming should be taken into account [75,76]. In such a scenario, the decreased viability of macrophages may impact patient recovery. Overall, the tested venom exerted some effects against cancerous cell lines; hence, it would be interesting to investigate the effects of individual toxins to potentially derive novel anti-cancer agents.

While our study provided some novel insights into the composition and activity of *Naja nivea* venom, several limitations need to be considered. Firstly, our analysis is based on a shotgun approach, which is a powerful tool to unveil the molecular diversity of venoms. However, unfortunately, this method does not allow a quantitative analysis and, hence, abundances of toxins cannot be provided. Second, we worked with a commercially purchased pooled venom sample; hence, we were unable to further investigate the effects of venom variation. Lastly, our selection of cell lines for functional analyses is limited. In order to understand better the effects of *Naja nivea* venom on cytotoxicity, local effects, and long-term consequences, a broader set of cell lines and animal models should be employed [77,78]. We recommend that future studies on *Naja nivea* venom incorporate these considerations to gain deeper insights into the matter.

## 4. Conclusions

Snakebite envenomation is as a neglected tropical disease as well as a global medical burden. Detailed analysis of the venom systems of medically important snakes is needed to develop effective antivenoms and other treatments. We used a shotgun proteomics approach and 1D-SDS-PAGE profiling to increase the current understanding on the molecular diversity of Cape cobra venom, which is among the most potent venoms of the African “true” cobras (genus *Naja*). We identified a total of 156 venom components belonging to 17 distinct protein families and showed that the venom profile of *N. nivea* is rich in proteins of lower molecular weight. The major venom components were 3FTxs, one-third of which were neurotoxins and two-thirds cytotoxins. Several of the neurotoxic 3FTxs were α-neurotoxins, which are highly toxic in humans. Additional toxins such as members of CRISP, LAAO, and svMP were also identified. In line with previous studies and with what has been reported for some other non-spitting *Naja* species, few PLA_2_ proteins were found in our dataset. However, the results of our bioactivity profiling suggest that *N. nivea* venoms can display considerable PLA_2_ activity. Future work should therefore evaluate multiple populations of these species to account for dynamic intraspecific variability in venom composition.

## 5. Materials and Methods

### 5.1. Origin of Sample Material

Crude *N. nivea* venom was obtained from a commercial supplier (Latoxan, Portes lès Valence, France). The venom was collected from their captive stock animals stemming from a South African population (unknown locality and pool size) and lyophilized before shipment. The sample was stored at −20 °C before use.

### 5.2. One-Dimensional SDS-PAGE

To generate insight into the overall protein landscape within the *N. nivea* venom, we applied a one-dimensional sodium dodecyl sulfate–polyacrylamide gel electrophoresis (SDS-PAGE) as described previously [79]. Therefore, we dissolved the venom in ddH_2_O and Laemmli buffer toward final concentrations of 5 µg and 2 µg. For SDS-PAGE under reducing conditions, the mix contained 5% of 2-mercaptoethanol. Analysis under non-reducing conditions was lacking 2-mercaptoethanol. Next, samples were incubated at 95 °C for 5 min and then loaded on 4–20% Mini-PROTEAN^®^ TGX™ Precast Protein Gels (Bio-Rad, Hercules, CA, USA). Electrophoresis of the samples and the used protein size marker (Precision Plus Protein All Blue Standard 10–250 kDa, Bio-Rad) was carried out at 150 V for 60 min in a Mini-PROTEAN Tetra Vertical Electrophoresis Cell (Bio-Rad). Gels were stained by ROTI BLUE quick solution (Carl Roth, Karlsruhe, Germany) and destained with ddH_2_O. 

### 5.3. Tryptic Digestion

The shotgun proteomics strategy was based on a protocol established for other animal venoms [80,81]. Briefly, 10 μg of venom was dissolved in 25 mM ammonium bicarbonate solution containing 0.6 µM ProteaseMax (Promega, Madison, WI, USA). Disulfide bonds were reduced by 5 mM dithiothreitol during 30 min of incubation at 50 °C. Next, free cysteines were alkylated via 10 mM iodoacetamide for 30 min at 24 °C. The addition of excess cysteine was used to quench the reaction. Afterwards, trypsin was added (50:1) and the sample was digested overnight at 37 °C. The reaction was then inhibited through the addition of 1% trifluoroacetic acid. Purification of the sample was carried out through a C18-ZipTip system (Millipore, Burlington, MA, USA), and the generated tryptic peptides were vacuum-dried and subsequently redissolved in 10 μL of 0.1% trifluoroacetic acid. 

### 5.4. Mass Spectrometry

Following digestion and purification, the tryptic peptides were subjected to high-performance liquid chromatography on an UltiMate 3000RSLCnano device (Thermo Fisher Scientific, Waltham, MA, USA) for separation and decomplexation. We therefore injected 1 μg of the peptide solution into a 50 cm μPAC C18 column (Pharma Fluidics, Bath, UK) dissolved in 0.1% formic acid at 35 °C. A linear gradient of 3–44% acetonitrile over 240 min was used and column washing was performed with 72% acetonitrile with the flow set to 300 nL/min. Eluting peptides were analyzed in an Orbitrap Eclipse Tribrid MS (Thermo Fisher Scientific) linked to an Advion TriVersa NanoMate electrospray ionization system (Advion BioSciences, Ithaca, NY, USA) running in positive ionization mode with the spray voltage set to 1.5 kV at 250 °C. Scanning was performed in data-independent acquisition mode with a scanning time of 3 s and mass-to-charge ratio (*m/z*) range of 375–1500 with a resolution of 120,000. Auto-gain control was set to standard and a maximum injection time of 50 ms was applied. Most intense ions of each cycle with a threshold count >50,000 and charge states of 2–7 were selected with an isolation window of 1.6 *m/z* for higher-energy collisional dissociation (normalized collision energy = 30%). Fragment spectra were acquired in the linear ion trap using a rapid scan rate and normal mass range. The maximum injection time was set to 100 ms and selected precursor ions were excluded for 15 s following fragmentation.

### 5.5. Data Analysis

Data acquisition and analysis were carried out in Xcalibur v4.3.73.11 and Proteome Discoverer v2.4.0.305 (Thermo Fisher Scientific). Protein identification using MASCOT v2.6.2 and the UniProt “Serpentes” database (3 June 2021; 117,084,901 residues; 274,620 sequences) served as a repository for peptide searches with the following settings: precursor ion mass tolerance = 10 ppm, carbamidomethylation as global modification, methionine oxidation as variable modification, and one missed cleavage allowed. The fragment ion mass tolerance during linear ion trap MS2 detection was 0.8 Da, and the false discovery rate was limited to 0.01 in a decoy database. For qualitative analysis, we only considered proteins that were identified with a MASCOT score of at least 50, a false discovery rate (FDR) confidence of “high”, and at least two verified peptides. A comprehensive list of all identified venom components, their characteristics, and annotations is provided in Table S1. The raw proteomic data were uploaded to PRIDE under the ID PXD036965.

### 5.6. Cytotoxicity Assays

Cytotoxicity assays on venom from *N. nivea* were performed using several cell lines. We initially tested the human alveolar basal epithelial cell line A549 (CLS Cell Lines Service, Eppelheim, Germany) [82]. Briefly, cells were placed in 96-well plates and grown until reaching confluence. Venom was diluted in water, ionomycin (7.74 mg/mL stock, Cayman Chemicals, Ann Arbor, USA) was uptaken in DMSO, and cells were treated with either venom (0.25, 2.5, 25 µg/mL), ddH_2_O (negative control), or ionomycin (100 µM, positive control). Next, they were incubated at 37 °C under 5% CO_2_ atmosphere. After 48 h, cell viability was determined by photometrically quantifying the ATP content via the CellTiter-Glo Luminescent Cell Viability assay (Promega) following the manufacturer’s instructions. 

Additional cytotoxicity assays were undertaken on human embryonic kidney cells (HEK 293T), neuroblastoma cells (SH-SY5Y), and RAW 264.7 macrophages and peripheral blood mononuclear cells (PBMCs), following the protocol described by Erkoc and colleagues [83,84]. Cell viability was determined using the OranguTM assay (Cell Guidance Systems, Cambridge, United Kingdom). Therefore, 2 × 10^5^ cells from each tested cell line were placed in 96-well plates and exposed to different venom concentrations or ddH_2_O as a control for 24 h. Subsequently, 10 μL of OranguTM cell-counting solution was added, followed by 60 min of incubation. Absorbance was measured at λ = 450 nm with a wavelength of λ = 650 nm using an EnSpire 2300 Multimode Plate Reader (Perkin Elmer, Waltham, MA, USA). Celecoxib (100 µM) and ddH_2_O were applied as the positive control as negative control, respectively. Readings were normalized to the vehicle (ddH_2_O) and set to 100% cell viability. For every assay, 1 μL of treatment (i.e., venom, positive control, or negative control) was added to 99 μL of medium present in each well of the 96-well plates used. All experiments were carried out in triplicates.

### 5.7. Protease Activity Assay

To understand the proteolytic potential of the tested venom, we employed a photometric general protease activity assay. Protease activity was determined via a Protease Assay Kit (539125, Sigma-Aldrich, St. Louis, MO, USA), as described earlier [85]. Venom at three different concentrations (50, 100, 200 μg/mL) was tested against trypsin and ddH_2_O controls. In a 96-well plate, 25 μL of fluorescein thiocarbamoyl-casein derivatives (FTC-casein) was mixed with 25 μL of incubation buffer and 10 μL of either venom or control and incubated (37 °C, 220 rpm for 2 h). Then, 120 μL of 5% trichloroacetic acid solution (Carl Roth) was added prior to incubation for another 20 min. This was followed by 15 min of centrifugation at 4 °C and 500× *g*. Next, 40 μL of supernatant was transferred to a flat-bottom 96-well plate and then mixed with 160 μL of assay buffer. Absorbance was detected at λ = 492 nm on a BioTek Eon microplate reader using the Gen v2.09 software. The absorbance values from each treatment were normalized to positive controls (set at 100%), after subtracting the values from negative controls (set at 0%). All experiments were carried out in triplicates.

### 5.8. Phospholipase A2 Activity Assay

Phospholipase A_2_ (PLA_2_) activity was measured via the EnzChek™ Phospholipase A_2_ Assay Kit (E10217, Thermo Fisher), as described earlier [79]. We prepared the Lipid Mix by adding 30 μL of 10 mM dioleoylphosphatidylcholine (DOPC) to 30 μL of 10 mM dioleoylphosphatidylglycerol (DOPG), and 30 μL of 1 mM PLA_2_ substrate. We then added 5 mL of 1 × reaction buffer. To prepare 5 mL of substrate-liposome, we slowly injected 50 μL of Lipid Mix into the side of the vortex in the beaker using a pipettor fitted with a narrow orifice gel-loading tip. Subsequently, 50 μL of the controls and 50 μL of each venom sample at different concentrations (3.125, 6.25, 12.5, 25, and 50 μg/mL) were injected into wells of a black 96-well plate, and 50 μL of the substrate-liposome mix was added. The plate was in the dark incubated at room temperature for 50 min. Fluorescence was measured on a Synergy H4 microplate reader (BioTek) equipped for excitation at λ = 470 nm and fluorescence emission at λ = 515 nm. Measurements were normalized against the positive control (100%) after subtraction of the negative control (0%). All experiments were carried out in triplicates.

### 5.9. Assessment of Intracellular Calcium (Ca^2+^) Levels

We measured Ca^2+^ release within cells as described previously [83,84]. Briefly, 2 × 10^4^ HEK 293T cells were placed in 96-well poly-D-lysine-coated plates and incubated (37 °C, 24 h). Then, they were incubated with 4.19 µg/mL of Fluo-8-AM in Hanks’ balanced salt solution (HBSS) for another 1 h at 37 °C. Next, the Fluo-8/HBSS was replaced with 100 µL of fresh HBSS. Images were acquired using an ImageXpress Micro confocal high-content imaging system (Molecular Devices, Workingham, United Kingdom) with five frames per second. For the induction assay, cells were treated with a vehicle (negative control), *N. nivea* venom at three different concentrations (0.25, 2.5, 25 µg/mL), or 5 µM ionomycin (positive control). In the inhibition assay, 5 µM ionomycin was added to the venom-treated cells after 30 min. For both assays, images were periodically taken every second for 20 s. Analysis of data was performed with the software MetaXpress v. 3.1.0.65, using a fluorescence intensity threshold established from pre-treatment cells. Cells exceeding this threshold were quantified. In the induction assay, the number of cells surpassing the threshold in venom-treated samples was compared to untreated samples. For the inhibition assay, venom-treated samples were compared to ionomycin-treated cells. Data were normalized to the positive control (100%) for the induction assay, and to the vehicle/negative control (100%) for the inhibition assay. All experiments were carried out in triplicates.

### 5.10. Assessment of Nitric Oxide (NO) Levels

Effects of *N. nivea* venom on the synthesis of NO were assessed following the method described by Erkoc and colleagues [83,84]. RAW 264.7 macrophages were seeded at a density of 2 × 10^4^ cells in a 96-well plate and incubated at 37 °C for 24 h. Then, *N. nivea* venom at varying concentrations (0.25, 2.5, 25 µg/mL), vehicle (negative control), or 0.1 µg/mL of lipopolysaccharide (LPS, positive control) was added. Alternatively, cells were pre-treated with venom or vehicle for 30 min before being exposed to 0.1 µg/mL of LPS to evaluate the inhibition of NO production. After 24 h of incubation, supernatants were collected and stored at −80 °C. A standard curve was established with sodium nitrite concentrations ranging from 0 to 3.45 µg/mL. For the assay, 80 µL of either cell supernatant or standard was mixed with 20 µL of sulfanilamide solution (40 mg/mL in 1 M HCl) and 20 µL of naphthalene diamine solution (60 mg/mL of N-(1-naphthyl)ethylenediamine dihydrochloride in water), followed by a 15 min incubation. Absorbance was quantified at 540 nm using an EnSpire Plate Reader (Perkin Elmer). Data from the induction and inhibition assays were normalized to 100% based on the positive control and vehicle/negative control, respectively. All experiments were carried out in triplicates.

## Figures and Tables

**Table 1:** Overview of the 3FTxs found in the analyzed *Naja nivea* venom. The identified 3FTxs were classified into the known subgroups and listed with the respective Uniprot ID, taxonomic origin, and MASCOT score for each hit identified via proteomics.

Subgroup	Uniprot ID	Taxon	MASCOT Score	Subgroup	Uniprot ID	Taxon	MASCOT Score
**Cytotoxins**	P01453	*Naja annulifera*	39,445	**Cytotoxins (*cont*.)**	P62390	*Naja annulifera*	1525
	P01456	*Naja nivea*	37,149		P01446	*Naja kaouthia*	1314
	P01463	*Naja nivea*	25,422		O93472	*Naja sputatrix*	1314
	P01462	*Naja annulifera*	24,579		P01474	*Naja melanoleuca*	673
	P01461	*Naja annulifera*	10,362		P60308	*Naja atra*	325
	P01458	*Naja nivea*	10,320	**Short Neurotoxins**	P01423	*Naja nivea*	5245
	Q98961	*Naja atra*	9746		P01421	*Naja annulifera*	2871
	P60311	*Naja sputatrix*	9574		P01422	*Naja annulifera*	2798
	O73857	*Naja sputatrix*	9574		P01426	*Naja pallida*	1929
	P01451	*Naja oxiana*	9574		P01424	*Naja melanoleuca*	1225
	O93473	*Naja sputatrix*	9572		Q9W717	*Naja atra*	866
	A0A0U5ARS4	*Naja naja*	9572	**Long Neurotoxins**	P01390	*Naja nivea*	47,565
	A0A0U4W6H0	*Naja naja*	9572		R4G2D8	*Acanthophis wellsi*	1388
	P01457	*Naja haje*	9446		Q53B57	*Ophiophagus hannah*	84
	P01468	*Naja pallida*	9321	**Muscarinic Toxins**	P60234	*Dendroaspis angusticeps*	669
	P01469	*Naja mossambica*	9321	**Non-conventional**	P25680	*Naja nivea*	7648
	P0DSN1	*Naja nigricollis*	8467		P01400	*Naja melanoleuca*	1554
	P86540	*Naja naja*	8281		P01401	*Naja haje*	1539
	P83345	*Naja sagittifera*	8275		P25677	*Naja annulifera*	902
	Q98956	*Naja atra*	8275		P29181	*Naja naja*	596
	P14541	*Naja kaouthia*	1567		P81782	*Bungarus candidus*	317

**Table 2:** Venom diversity comparison of five studies, including the dataset presented here, on *N. nivea* venom proteomes. Toxin families: three-finger toxins (3FTx), cytotoxins (CTx), neurotoxins (NTx), non-conventional 3FTx (NC), muscarinic toxins (MTx), snake venom metalloproteinases (svMP), snake venom serine proteases (svSP), aminopeptidases (AP), cysteine-rich secretory protein (CRISP), l-amino acid oxidase (LAAO), 5′-nucleotidase (5N), phosphodiesterase (PDE), carboxylesterase (CE), phospholipase A_2_ (PLA_2_), phospholipase B (PLB), nerve growth factor (NGF), Kunitz-type inhibitor (KUN), vascular endothelial growth factors (VEGF), hyaluronidases (HYAL), cystatin (CYS), C-type lectins and lectin-related proteins (CTL), and disintegrins (DI). Abbr.: mass spectrometry (MS), area under the curve of the extracted ion chromatograms (AUC XIC), label-free quantification (LFQ), mean spectral intensity (MSI), and normalized spectral abundance factor (NASF).

Toxin Family and Subgroups	This Study	Kazandjian et al., 2021	Nguyen et al., 2022	Tan et al., 2022	McFarlane et al., 2024
**3FTx**	✓	✓	✓	✓	✓
- CTx	✓	✓	✓	✓	✓
- Short NTx	✓	✓	✓	✓	✓
- Long NTx	✓	✓	✓	✓	✓
- NC NTx	✓	✓	✓	✓	✓
- MTx	✓		✓		
- Other 3FTx subgroups			✓		
**svMP**	✓		✓	✓	✓
**svSP**	✓		✓		✓
**AP and other peptidases**	✓		✓		
**Venom Complement C3**	✓		✓	✓	✓
**CRISP**	✓	✓	✓	✓	✓
**LAAO**	✓		✓	✓	✓
**5N**	✓		✓	✓	✓
**PDE**	✓		✓	✓	✓
**CE and other esterases**	✓		✓	✓	✓
**PLA_2_**	✓	✓	✓		✓
**PLB**	✓		✓		
**Endonuclease**	✓				
**NGF**	✓	✓	✓	✓	✓
**KUN**	✓	✓	✓	✓	✓
**Vespryn**	✓		✓	✓	✓
**VEGF**	✓		✓		
**HYAL**			✓		✓
**CYS**			✓		
**CTL**			✓		
**DI**			✓		
**Other proteins and peptides**			✓		✓
**Proteomics workflow**	Shotgun	Top-down	Shotgun	Bottom-up	Bottom-up
**(Semi)-quantification**	No. of toxins	MS (AUC XIC)	MS (LFQ)	RP-HPLC + MS (MSI)	MS (NASF)

## Data Availability

Proteomic raw data files have been uploaded to the open access PRIDE database (PXD03696). All identified venom components are described in Table S1. Crude venom samples are available from the corresponding author upon reasonable request.

## Supplementary Materials

The following supporting information can be downloaded at https://www.mdpi.com/article/10.3390/toxins16100438/s1. Figure S1: Additional SDS-PAGE gel with increased venom concentrations; Table S1: Full results of the proteomic analysis performed on the analyzed *Naja nivea* venom; Table S2: Raw data of the cytotoxicity assays performed on A549 cells; Table S3: Raw data of the cytotoxicity assays performed on PBMC, RAW 264.7, HEK 293T, and SH-SY5Y cells; Table S4: Raw data of the protease activity assay; Table S5: Raw data of PLA2 activity assay; Table S6: Raw data of assessment of intracellular Ca^2+^ release; Table S7: Raw data of assessment of NO levels.
