# Supplementary figures and images for: The Toxin Diversity, Cytotoxicity, and Enzymatic Activity of Cape Cobra (Naja nivea) Venom
Source: Toxins (Basel). 2024 Oct 11;16(10):438. doi: 10.3390/toxins16100438 (PMC11511112; doi:10.3390/toxins16100438)

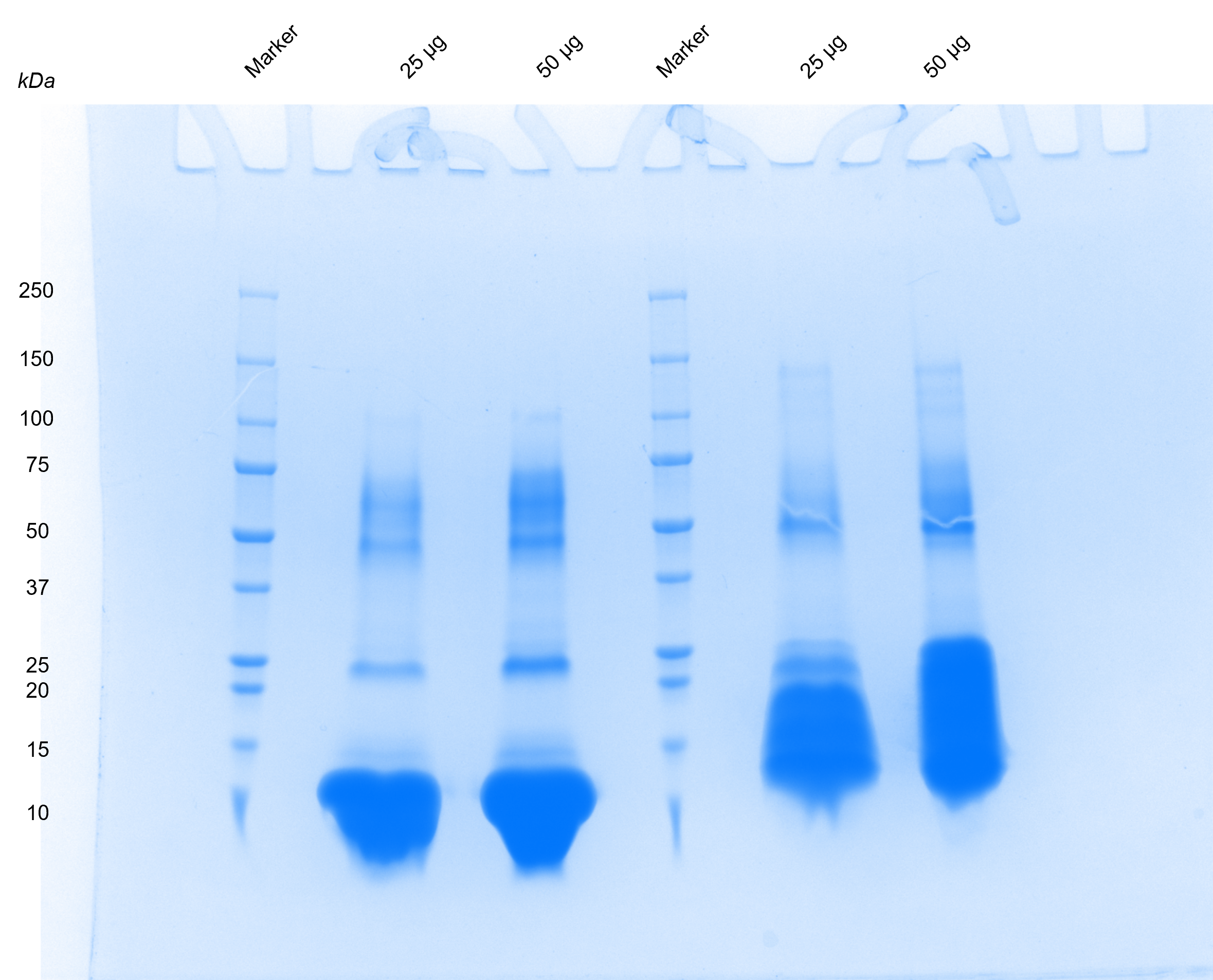

Supplement: Supplementary file 1 [file toxins-16-00438-s001.zip › Supplementary figure S1.png]
